# Mentoring for INnovative Design Solutions (MINDS): Key Design Considerations and Collaborative Teamwork across Universities for Clinical Translation

**DOI:** 10.1007/s43683-022-00090-3

**Published:** 2022-11-28

**Authors:** Alicia Fernandez-Fernandez, Walter Lee Murfee, Jeffrey A. LaMack, Teresa A. Murray

**Affiliations:** 1 Physical Therapy Department, Nova Southeastern University, Ft. Lauderdale, FL USA; 2 J. Crayton Pruitt Family Department of Biomedical Engineering, University of Florida, Gainesville, FL USA; 3 Electrical Engineering and Computer Science Department, Milwaukee School of Engineering, Milwaukee, WI USA; 4 Center for Biomedical Engineering and Rehabilitation Sciences, Louisiana Tech University, Ruston, LA USA

**Keywords:** Experiential learning, Extracurricular learning, Professional development, Medical device design, Interinstitutional collaboration, Mentorship

## Abstract

The main purpose of this paper is to share the Mentoring for INnovative Design Solutions (MINDS) Scholars Program developed by Alpha Eta Mu Beta, the International Biomedical Engineering Honor Society. The program’s goals are to (1) introduce biomedical engineering students to an open-ended design experience as part of interuniversity teams with industry and faculty mentors, and (2) develop the ability to create designs considering clinical translatability on teams with different backgrounds and areas of expertise. MINDS uses an experiential learning approach to (1) enrich student curricular experiences through inter-institutional collaboration, (2) build engineering design skills, including three key design considerations for clinical/commercial success: intellectual property protection, regulatory strategy, and market identification; and (3) emphasize the importance of end-user considerations. From 2015 to 2022, MINDS has involved 131 students from 50 universities and 22 faculty and industry mentors. Pre- and post-program surveys show statistically significant improvements in understanding of the design process, regulatory strategy, intellectual property protection, market definition, and key product requirements and features. Students also improved communication and teamwork skills. Many students indicated that MINDS participation made them more likely to choose careers that involve product development and/or entrepreneurship. Students attained a working ability to integrate market needs, regulatory strategy, and intellectual property considerations into the design process. They also further developed soft skills, such as conflict resolution, time management, and effective communication through the challenges of inter-institutional collaboration. Additionally, the program heightened their awareness of how biomedical devices and technologies can benefit society.

## Introduction

Experiential learning (EL) enhances the depth to which engineering students grasp technical concepts and their ability to apply learned concepts to solve new real-world problems.^1^ It uniquely involves participation of the learner in an activity from which they are guided to reflect and conceptualize to put them into a position to apply what is learned to new situations.^1–3^ Biomedical engineering (BME) presents many opportunities for EL in the curriculum, as there is a rich array of real-world problems in healthcare demanding technical solutions that can be brought into the classroom. EL activities have been incorporated as early as first year introduction into biomedical engineering courses,^4^ as well as courses later in the curriculum, and in capstone design.^5^ For such experiences, the emphasis tends to focus on technical problem solving, as well as critical elements of the engineering design process, including problem and needs identification.

Considering the impacts of EL, opportunities exist to apply EL to advanced-level engineering skills to address gaps in BME curricula that often challenge student preparedness. First, student design experiences may or may not provide students with the opportunity to learn about important elements of commercializing medical device solutions. Yet there continues to be demand in industry for undergraduates who understand basic principles of medical device regulation, intellectual property, and market considerations for medical devices.^6^ Also, additional important professional skills that are recommended as areas of emphasis in BME curricula are communication, presentation, and teamwork skills, and there is a recognized need to expand coverage of these skills beyond senior level capstone courses.^7^ Finally, the historic lack of consensus of the BME identity has led to inconsistent student perceptions of what it means to be a biomedical engineer, particularly in the context of becoming employed.^8^ Potential solutions for covering BME curricular gaps can involve student and professional organizations.

In a recent review of EL in undergraduate engineering education, Jamison et al. noted a specific gap in research on impacts of experiential learning models in co-curricular and extra-curricular learning contexts, noting that the majority of work in this area has been on topics addressed in the classroom.^1^ These findings further support the opportunity for filling in BME curricula gaps through organizationally-initiated EL experiences. Student organizations provide an extra-curricular context for experiential learning to contribute to professional development, including leadership, communications, and professional identity.^9^ Additionally, involvement in professional engineering organizations improves rates of persistence and identity development of underrepresented and minority students in engineering curricula by providing networking opportunities, access to professional resources, and reduction of perceptions of isolation based on gender or race/ethnicity.^10^ A next leading approach would be for organizations to integrate EL programs to further enhance student preparedness.

Recognizing the benefits of extra-curricular EL programs, as well as the potential for such programs to develop professional skills of value for BME students, Alpha Eta Mu Beta (AHMB) developed the Mentoring for INnovative Design Solutions (MINDS) Scholar Program in 2015. AHMB is the international biomedical engineering honor society. It was founded in 1979 and now oversees student chapters at more than fifty universities. Over the past seven years, AHMB has evolved the program to introduce BME students to advanced engineering skills by guiding collaboration across different universities during an open-ended design experience. Guided by mentors from academia and industry, students work in teams to identify a problem in healthcare, formulate stakeholder needs for a potential solution, and develop a conceptual solution while considering the market, intellectual property potential, and regulatory requirements for the product. Teams work using virtual communications over the course of up to 6 months to complete a series of deliverables culminating in a final presentation of their design concept. This paper describes the objectives, logistics, outcomes, and lessons learned from the MINDS program between 2015 and 2022.

## Methods

### Program Overview

The MINDS program EL environment is used to (1) enrich student curricular experiences through inter-institutional collaboration, (2) build engineering design skills, including extensive integration of three key considerations into their design which are often not as well-developed in coursework: intellectual property protection, regulatory strategy, and market identification; and (3) emphasize the importance of end-user considerations in a successful design. Students also build communication skills and are exposed to additional career pathways. Participants are selected based on a competitive application process and they are placed into diverse teams (geographic, class level, gender, etc.) with 2 mentors per team. Teams meet and begin working together during an initial guided workshop. After this meeting, they continue to collaborate virtually for up to 6 months to refine their designs, with support from mentors and outreach to other experts. Incremental “mini-deliverables” guide students from a basic problem description, through increasingly detailed specifications and design choices, to the final design and project report. Students and mentors are surveyed at the beginning and end of the program under an IRB-approved protocol. The information is utilized for continuous improvement of the program by the MINDS Steering Committee which is composed of AHMB officers and Board members.

### Program Objectives and Philosophy

Program objectives (Table 1) were designed to supplement traditional engineering curricula by providing students with exposure to aspects of the design process that are not traditionally emphasized in coursework, and which may be only introduced or covered with reduced breadth and scope as part of senior design classes. Additionally, the objectives also encompass the development of important soft skills related to communication, collaboration, and working in diverse teams, which are critical in today’s changing professional landscapes. By extension, successful completion of these objectives also requires skills in asynchronous work, time management, and use of technologies, all of which have become indispensable not only in engineering but across many sectors of the workforce.

**Table 1:** Student learning objectives for MINDS program.

The goal of the MINDS program is for participating students to achieve the following learning objectives by the end of the program:
1. Manage a collaborative long-term project with teammates and mentors from different institutions.
2. Formulate a list of key design requirements to address a healthcare problem and design a conceptual medical device solution to meet those requirements.
3. Identify the market and evaluate the potential for commercialization of a medical device solution concept.
4. Evaluate existing intellectual property to determine patentability of a specific design idea.
5. Develop and defend a regulatory strategy and pathway to market for a novel medical device concept.
6. Communicate technical information effectively in oral and written formats.

The philosophy of the program is fundamentally experiential and focused on how learning can take place outside the classroom in a guided, structured but at the same time open, adaptive manner. Students are provided with a framework that allows them to learn by doing and that pushes them to explore different areas that are mostly unfamiliar to them. Mentors guide and mold this discovery journey to maximize learning, while allowing students to figure out their own path. Teams are given a general prompt for a design idea that allows them a considerable degree of freedom to choose their topic area, and then they are provided with checkpoints along the way to promote accountability and steady progress. The program strives to achieve a balance between supporting students and allowing them to be responsible for their learning. Projects are student-driven, and each team collaboratively decides on a project topic of their choosing. They refine and define a problem statement based on their chosen topic, with input and guidance from their mentors. Problem ideas are usually based on the ideas written in their program application, but this is not a requirement. An important note is that teams are primarily formed based on the integration of students across universities and academic levels versus ideas.

### Selection of MINDS Scholars and Formation of Teams

Students are selected in a nationally competitive process. Beginning in late summer, emails that describe the MINDS Scholars Program and the application process are sent to Biomedical Engineering and Bioengineering department chairs across the US and to AHMB chapter advisors. A FAQ sheet is also sent for posting where students will see the information.

Students must complete an application for consideration. Membership in AHMB is not a requirement for acceptance. In their applications, students identify an unmet need for a biomedical device or technology (innovation) and its potential impact. The general theme for the MINDS Program is “Biomedical Engineering Design for Unmet Health Care Needs”, which provides ample freedom for students to select a topic area of their interest. Applicants are judged on the quality and novelty of the design idea and their potential impact statement. They also write a brief essay describing their interest in participating in the MINDS program and their related experiences, if any. Scholars are evaluated independently by three MINDS Steering Committee members who use a rubric to assign scores to each application. Students with the highest average scores are considered for the program. The statements on related experiences, if any, are used to ensure that teams have at least two students with some project/design experience that can be peer mentors to those with little or no such experience. The students’ design ideas are used to group students with related interests into teams of three to four students who are then matched with two mentors. Gender, institutional, and geographical diversity are also established when assigning students to teams.

One of the key aspects of the MINDS program is working with peers and mentors from different institutions, and this has been emphasized in the most recent cohorts of the program. Introducing students to inter-institutional collaboration provides repeated experiences for students to improve their communications skills and discipline to adhere to schedules, especially when members are in two or more time zones. Each team has two mentors. The primary mentor helps the students navigate the ideation process and the key design considerations. This mentor also provides a point of accountability for students to stay on their timeline for deliverables. The secondary mentor complements the primary mentor’s perspective and background during the ideation and refinement processes, increasing the resources for students, and ensuring that at least one mentor is available during team meetings. Sometimes, students need specialized expertise. Mentors use their professional networks to help students connect with experts.

### Funding

Conference grants from the US National Science Foundation have provided the funding required to provide travel grants for students to attend the initial MINDS Workshop as well as defray a portion of the costs for hosting the event. The Workshops have been funded in this manner from 2016 to the present.

### Program Components

**Figure 1:**
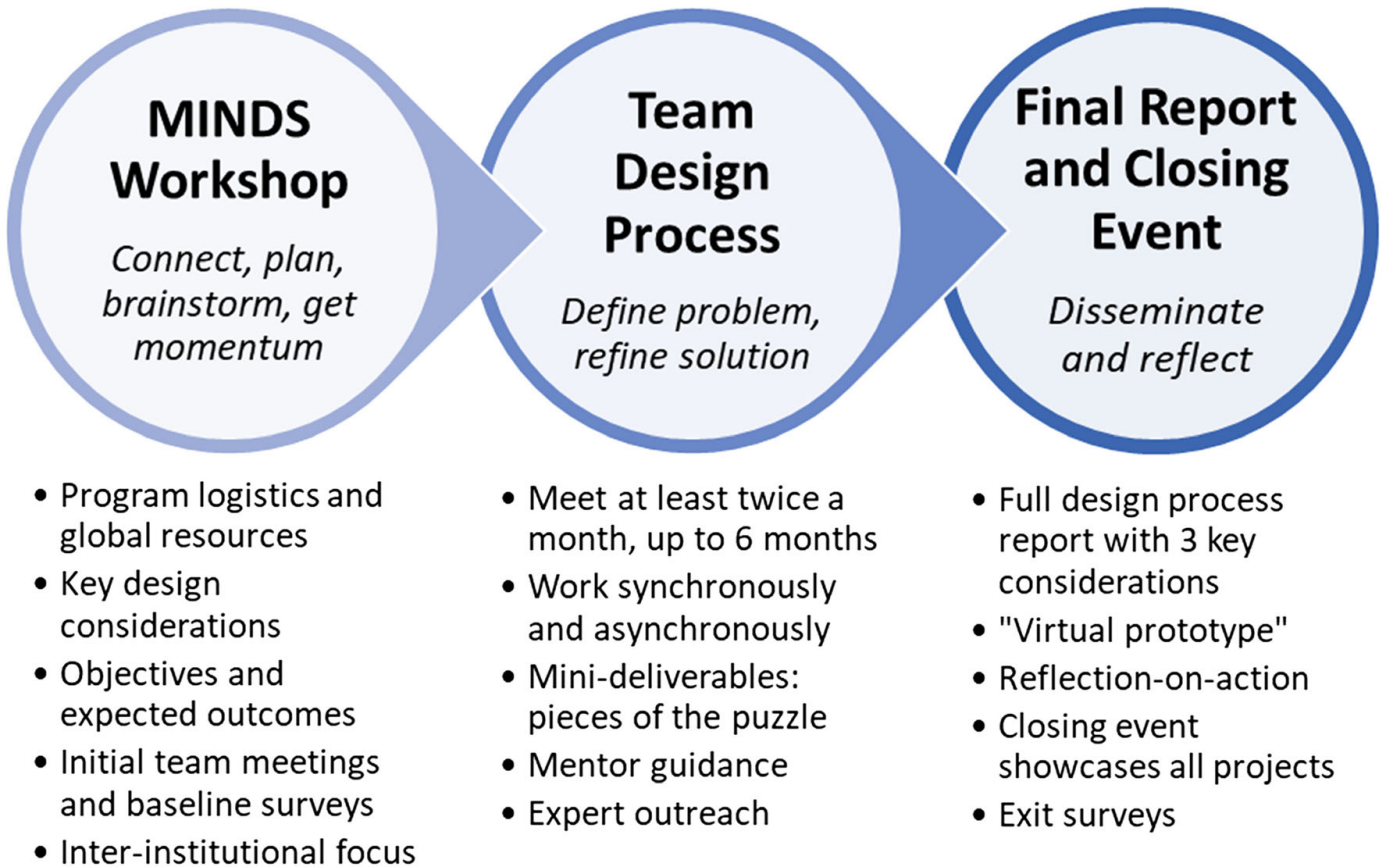
MINDS program timeline and components.

An overview of the components of the MINDS program is illustrated in Fig. 1, and subsequent subsections provide details for each component.

### MINDS Workshop

The MINDS workshop that initiates the MINDS program is typically timed with the Biomedical Engineering Society (BMES) Annual Meetings. Due to the COVID-19 pandemic, the workshop was offered virtually in 2020 and 2021, and was coupled with virtual registration for the BMES Annual Meeting. The Scholars’ educational and professional development is further enriched by attending poster sessions and oral presentations as well as the conference’s networking events.

Each annual workshop lasts approximately 2 hours and begins with a brief welcome and an overview of the program, including timelines, expected deliverables, incorporation of the three key considerations into the design process, and use of the program’s online resources and team collaboration sites. Since 2018, there is also a short presentation from a previous MINDS Scholar who talks about their experience and gives some general tips about how to succeed in the program. Next, the group breaks into their preassigned teams of mentors and students to share schedules, communication preferences, interests, and plans for the program. The final portion of the team meeting is spent with mentors guiding an initial ideation process and discussions of the three key considerations for two or three of the design ideas. This process is not intended to result in a decision to proceed with a specific design. Rather, it is for students to immediately begin using some of the processes and tools that they will use and refine throughout the program.

During the team meetings, one or two facilitators circulate between teams ensuring that participants stay on schedule, helping them navigate online resources, and reporting back to the workshop leaders on the teams’ progress. At the conclusion of the workshop, a facilitator summarizes the progress made by teams, reminds participants where to find post-workshop resources, reviews the due dates of future deliverables, and provides a survey link. The survey assesses the knowledge gained from the workshop, its ability to stimulate ideas, and requests comments to improve the workshop. In this survey, participants are asked to write at least one main concept for each of the three key design considerations. Mentors are given a survey to evaluate the performance of the students and their apparent level of understanding of key concepts, the ability of the workshop to stimulate ideas, and to suggest improvements. All surveys and dissemination of the results have been approved as exempt by the Institutional Review Board of  Nova Southeastern University.

### Additional Resources

Information on the Food and Drug Administration (FDA) regulatory requirements, intellectual property protection, and basic marketing procedures are provided to participants and mentors to assist teams to more effectively develop their plans to address the program’s three key design considerations. This information is available in an online repository and includes tutorials, websites, and links to webinars; and is updated yearly as needed or as new resources become available. Students are also encouraged to attend any other BMES annual meeting sessions that may be related to their topic or to design considerations. In addition, the MINDS Steering Committee has produced and recorded topical webinars and in-person BMES conference sessions featuring experts in the fields of regulatory strategy, intellectual property protection, and marketing of medical devices to provide detailed information that is specific to the medical device and diagnostics field.

Initially, webinars on a specific topic, such as regulatory strategy or IP strategy, occurred after the BMES meeting during the first six weeks after the initial workshop. Students and mentors participated from their home institutions. Since participants could view the recorded sessions asynchronously, relatively few participated during the live event. In subsequent years, live workshops were held during the BMES Annual Meeting the day after the MINDS workshop. These sessions were open to all BMES Annual Meeting attendees, which more than doubled the number of students exposed to the content.

Live sessions and webinar speakers included industry professionals in regulatory affairs and intellectual property (IP) consultants and lawyers, and speakers with marketing and entrepreneurial experience. Each live session and webinar provided ample time for students to ask questions. All events were recorded so that students could review the content, as needed during the design process. The talks on regulatory strategy and IP protection were especially valuable. Together, they highlighted the challenges of striving to show the novelties of a medical device to obtain patents while satisfying the need to show substantial equivalence to an existing medical device to streamline the FDA approval process. Due to the implementation of COVID-19 restrictions in 2020 and 2021, the live session was canceled but students had access to recordings from previous years.

### Choosing a Design and Refinement

After the workshop at the BMES conference, students and mentors meet virtually for up to 6 months to finish the ideation process, choose a design, and then refine it. During these processes, Scholars use the three key considerations to produce a marketable product design that has the potential for FDA approval, patent protection, and a positive impact on the people it is intended to help. Based on early experiences with the program, it is recommended that teams meet twice per month; however, teams are allowed freedom to plan their schedule and project timeline. Scholars are encouraged to find subject matter experts to provide them with specialized knowledge in areas such as market evaluation, patenting devices, and determining regulatory pathways for their device. Sometimes, advice from an expert in a particular field of manufacturing or medicine will help a team improve its design or make it more relevant for a particular patient population. If needed, mentors provide direction on where students can find experts and how to ask for assistance.

Teams are given a set of mini-deliverables with deadlines. Mentors remind students about deliverables, if warranted. Students are responsible for submitting each deliverable to the project’s online repository. Originally, larger components (milestones) were achieved over several weeks or months. Student and mentor feedback suggested that this long-term model could become overwhelming for the teams. The mini-deliverables reduce large project components into smaller, more easily achieved tasks which are due more frequently. Examples of deliverables include prompts such as “describe your problem” (summarize the problem, describe the need, the impact, and what a solution would need to do, along with a design "wish list"), “the three key design considerations” (preliminary analysis of intellectual property, regulatory strategy, and marketing considerations), or “virtual prototype” (design specifications with drawings, tables, etc. as applicable). Students have 3 or 4 weeks to complete each of the tasks and submit them.

### Design Reports

At the conclusion of the program, each team has a refined product design. Scholars create a report which can range from a traditional written report with tables and figures to a mock website or video promotion. All reports must show how Scholars used the key design considerations in their design process, how they have considered end-user needs, and their reflection on the learning process. Students are provided with report guidelines at the beginning of the program, which include all the sections that must be covered in the report and some guiding prompts for each section. This ensures that teams have a clear set of expectations of what they need to accomplish by the end of the program. At a minimum, the report must include: (1) Introduction, (2) Description of the design process, (3) Description of how the three design considerations (marketing, intellectual property, regulatory strategy) impacted the design, (4) Description of the proposed device or solution, including benefits and comparisons with existing technology, (5) End-user considerations, (6) Reflection, and (7) References.

### Closing Event

For the 2020–2021 cohort, a closing event/virtual design showcase was added after the due date of the final report which was held in April 2021. Prior to this cohort, project reports were shared online but there was no final interaction or discussion of the different projects between teams. Student feedback uniformly suggested that they would like to actively learn more about what other teams had done. The first closing event in 2021 was simply an informal gathering with opportunity for discussion, and no formal expectations were placed on the teams. Although this was meant to avoid putting additional pressure on participants after having turned in their final report, it also meant that there was high variability in attendance, as well as in the quality of what each team shared. For the 2021–2022 cohort, more structure was added in order to fully realize the vision of the closing event. Each team had ten minutes to describe their design with appropriate drawings, tables, and other visuals, followed by some time for questions and comments from peers and mentors. Students were very receptive to this format and excited about attending this event, sharing their work with others, and “graduating” from the program.

### Outcome Assessment

Besides the initial survey completed after the workshop, which focuses more on initial logistics, demographics, and the level of knowledge of incoming participants in terms of the design considerations, Scholars and mentors also complete an exit survey which is open for up to 3 weeks after submission of the final report. In this exit survey, students are asked to rate the perceived impact of the MINDS program on the key skills that are defined by the program learning objectives, including collaboration, design requirements, evaluation of market needs, evaluation of intellectual property considerations, development of a regulatory strategy, and communication of technical information. Questions on some of these items are broken down into more granular sub-skills when necessary, in order to refine survey data and allow for its use in continuous program improvement. For instance, students were asked about objective 2, “formulate a list of key design requirements” not as an overall question but rather as part of several survey items. These included, for example, ability to apply the 3 key design considerations, articulate key features of a product, or articulate key benefits and impact of a product. The wording of specific questions included in the survey is detailed in the results section along with the response analysis. Students were also asked to provide open-ended comments about program logistics, what they liked or disliked about the program, whether it had impacted their engineering skills or their career plans, and any other comments they wanted to make on their experience. Similarly, mentors were asked to rate student skills in the key areas, and to provide feedback on the program.

### Data Analysis

Data from surveys was imported into Excel for de-identification and to aggregate all cohorts. Quantitative data was then processed in SPSS 28 (Armonk, NY: IBM Corp) to create frequency tables and to compare pre-and post-program perceived knowledge in key areas. Comparison was done using Wilcoxon signed-rank tests due to the ordinal nature of the data. Multiple comparisons were accounted for with a Bonferroni correction (threshold alpha = 0.01).

Qualitative data analysis followed an inductive approach, where open-ended comments in the exit surveys were systematically analyzed using the constant comparative method. The analysis was guided by specific learning objectives as well as the characteristic components of experiential learning. Briefly, comments were extracted from the surveys and placed into categories determined by the key content of the comment in relation to learning objectives, target outcome skills, or the lived experience of experiential learning. Categories were cross-compared to determine connections into core categories and patterns, which were then built into major themes that summarized the overall intent of the related categories. Two initial rounds of coding were completed, with participation from all 4 authors to refine core categories, analyze overlaps, and build themes. A third round of discussion was utilized to ensure theoretical data saturation and finalize themes.

## Results

A total of 131 students successfully completed the MINDS program between 2015 and 2022. Collectively, they worked on 34 projects that spanned a multitude of areas chosen by the teams. Topic areas for projects from 2020 to 2021 and 2021–2022 are provided as a sample in Table 2. Student exit survey response rate was 96.2% (126 students out of 131 fully completed the exit survey).

**Table 2:** MINDS project topic areas from 2020 through 2022.

*2021-2022 Cohort* • Surgical Stapler for Colorectal Applications • Prenatal Detection of Congenital Heart Disease • Wearable Device for Stroke Detection • Self-Adjusting Prosthesis • Parkinson's Anti-Tremor Handle (PATH)
*2020-2021 Cohort* • Wrist Assist for Individuals with Disabilities • The 3PD: Point-of-Care Plasmon Prognosis Device • Covid-19 Rapid Toothbrush Test • Nanoparticle Face Mask Filters for Increased Efficacy and Safety

### Student Demographics

Demographic information on MINDS participants is shown in Table 3. Participants represented a diversity of class levels and ethnicities, and there was a similar percentage of males and females. MINDS Scholars came from 50 different universities, spanning all regions of the United States (Northeast, Southeast, Midwest, West, and Southwest), as well as two students from the University of Toronto in Canada.

**Table 3:** MINDS participant characteristics.

*Year (n = 126)*	
Freshmen	2 (1.6%)
Sophomores	35 (27.8%)
Juniors	61 (48.4%)
Seniors	26 (20.6%)
Graduate students	2 (1.6%)
*Ethnicity (n = 104)*	
American Indian or Alaskan Native	1 (1.0%)
Asian or Pacific Islander	32 (30.8%)
Black or African American	3 (2.9%)
Hispanic or Latino	7 (6.7%)
White	52 (50.0%)
Multiple ethnicities	9 (8.7%)
*Gender (n = 126)*	
Female	64 (50.8%)
Male	62 (49.2%)

### Mentor Demographics

A total of 22 different mentors have participated in the MINDS program between 2015 and 2022, from a total of 18 different institutions. The majority of them (72.7%) have mentored multiple cohorts, which contributes to continuity and consistency of the program. Of these mentors, 4 (18.2%) have their primary job position in industry, and 18 (81.8%) have their primary job position in academia; all mentors except for one have doctorate degrees (the remaining mentor has a master’s degree). Mentors came primarily from the Southeast and Midwest regions of the U.S. In the initial years, groups had one mentor each, but starting with the 2017–2018 cohort, a two-mentor model was implemented in response to student and mentor feedback that recommended having two mentors available to help with group logistics, as well as to expose students to a variety of backgrounds and expertise.

### Outcomes

**Figure 2:**
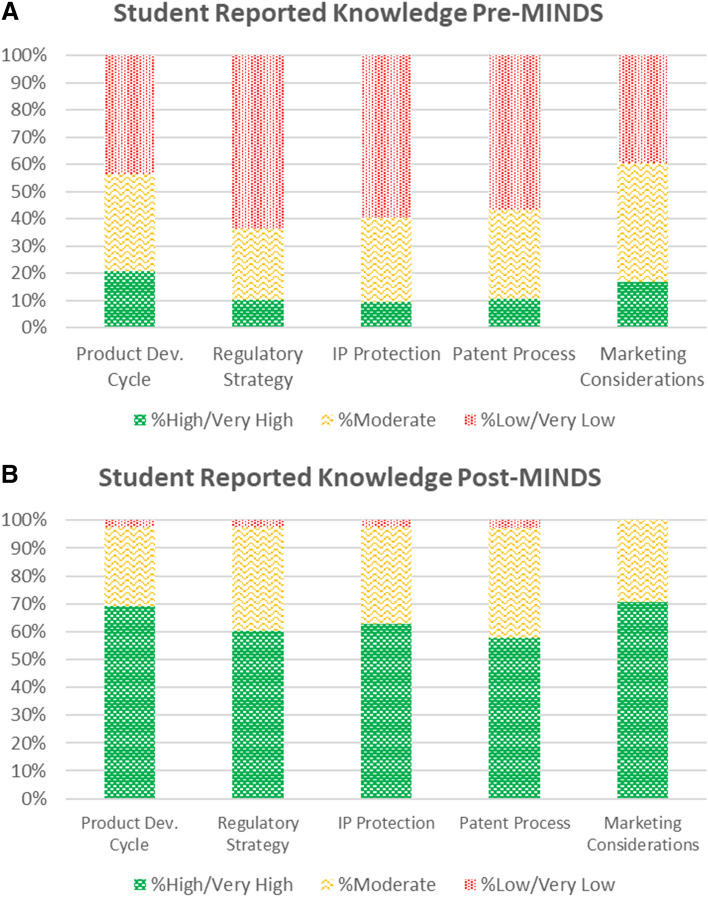
Student self-reported knowledge in key areas pre (A) and post (B) MINDS.

Student outcome surveys demonstrated that student self-reported knowledge increased significantly in all key content areas that are targeted by the program, including design process, regulatory strategy, intellectual property protection, the patent process, and marketing considerations (Table 4). As expected, students came into the program with a varied range of initial knowledge about these areas, but there was a clear shift towards increased self-perceived knowledge across the board after program completion. Figure 2 illustrates this shift in a more visual format.

**Table 4:** Student self-reported knowledge in key areas: pre- vs. post-MINDS.

	Before MINDS frequency (%)					After MINDS frequency (%)					Wilcoxon signed-ranks test	
	1 Very low	2 Low	3 Moderate	4 High	5 Very High	1 Very low	2 Low	3 Moderate	4 High	5 Very High	Standardized test statistic	*P*-value
Product development cycle/design process (n=126)	11 (8.7 %)	44 (34.9%)	45 (35.7%)	22 (17.5%)	4 (3.2%)	0 (0.0%)	3 (2.4%)	36 (28.6%)	62 (49.2%)	25 (19.8%)	9.532	< 0.001 *
Regulatory strategy (n=126)	30 (23.8%)	50 (39.7%)	33 (26.2%)	12 (9.5%)	1 (0.8%)	0 (0.0%)	3 (2.4%)	47 (37.3%)	53 (42.1%)	23 (18.3%)	9.889	< 0.001 *
Intellectual property protection (n=126)	32 (25.4%)	43 (34.1%)	39 (31.0%)	9 (7.1%)	3 (2.4%)	1 (0.8%)	2 (1.6%)	44 (34.9%)	58 (46.0%)	21 (16.7)	9.784	< 0.001 *
Patent process (n= 104)†	25 (24.0%)	34 (32.7%)	34 (32.7%)	10 (9.6%)	1 (1.0%)	2 (1.9%)	1 (1.0%)	41 (39.4%)	44 (42.3%)	16 (15.4%)	8.824	< 0.001 *
Marketing considerations (n=126)	14 (11.1%)	36 (28.6%)	55 (43.7%)	19 (15.1%)	2 (1.6%)	0 (0.0%)	0 (0.0%)	37 (29.4%)	62 (49.2%)	27 (21.4%)	9.902	< 0.001 *

^†^Patent question was added to survey starting with the 2017-2018 cohort; * Significant difference (p< 0.01 based on Bonferroni correction)

In the exit survey, students were also asked to evaluate the impact of the MINDS program on their skills in several areas related to the program learning outcomes, including not only specific design-related skills but also soft skills, such as communication or teamwork. The results are shown in Table 5. Over 90% of the students agreed or highly agreed that MINDS had improved their perceived skills in all the surveyed areas, with the exception of “resolve conflicts” (67.5% agreed or highly agreed) and “understand roles of key personnel in product development” (69.1% agreed or highly agreed). Open-ended comments revealed further information on these two areas. For “resolving conflicts”, several students expressed that their team worked well together and thus they did not feel the program impacted their ability to resolve conflicts. Therefore, it seems more likely that only students who had conflict within their teams felt strongly enough about this question to mark it high or low on the scale. Regarding “understand roles of key personnel”, survey comments reveal that student exposure to different personnel was highly dependent on how the team had networked, or whether they reached out to outside experts beyond their assigned mentors. Therefore, not all groups had the same experience in terms of exposure to different personnel, such as patent attorneys, regulatory affairs managers, etc. They all had exposure to basic general concepts of regulatory strategy, intellectual property, and marketing strategies, as demonstrated by the significant improvement in knowledge in these areas that was previously shown in Table 4. Finally, students were also asked if they believed MINDS had an impact on their career goals, and 65.1% of students agreed or highly agreed with this statement.

**Table 5:** Student Self-Reported Outcomes in Key Skills “The MINDS program has helped me to….”.

Skills	Frequency (%)					Combined % that agree/highly agree
	1 Highly disagree	2 Disagree	3 Neutral	4 Agree	5 Highly agree	
1. Apply the three key design considerations	0 (0.0%)	2 (1.6%)	5 (4.0%)	69 (54.8%)	50 (39.7%)	94.4%
2. Articulate key features of product	0 (0.0%)	2 (1.6%)	10 (7.9%)	64 (50.8%)	50 (39.7%)	90.5%
3. Articulate benefits and impact of product	0 (0.0%)	2 (1.6%)	9 (7.1%)	69 (54.8%)	46 (36.5%)	91.3%
4. Assess key product requirements	0 (0.0%)	4 (3.2%)	6 (4.8%)	70 (55.6%)	46 (36.5%)	92.1%
5. Collaborate effectively	1 (0.8%)	2 (1.6%)	9 (7.1%)	48 (38.1%)	66 (52.4%)	90.5%
6. Improve teamwork skills	1 (0.8%)	3 (2.4%)	7 (5.6%)	56 (44.4%)	59 (46.8%)	91.3%
7. Resolve conflicts effectively	2 (1.6%)	6 (4.8%)	33 (26.2%)	52 (41.3%)	33 (26.2%)	67.5%
8. Communicate effectively	0 (0.0%)	3 (2.4%)	9 (7.1%)	66 (52.4%)	48 (38.1%)	90.5%
9. Explain technical subject matters to others	1 (0.8%)	2 (1.6%)	8 (6.3%)	72 (57.1%)	43 (34.1%)	91.3%
10. Understand role of key personnel in product development	3 (2.4%)	17 (13.5%)	19 (15.1%)	58 (46.0%)	29 (23.0%)	69.1%
11. Understand intellectual property / patent process	0 (0.0%)	3 (2.4%)	8 (6.3%)	68 (54.0%)	47 (37.3%)	91.3%
12. Understand development process from idea to virtual prototype	0 (0.0%)	2 (1.6%)	9 (7.1%)	55 (43.7%)	60 (47.6%)	91.3%
13. Identify limitations of a product	0 (0.0%)	0 (0.0%)	5 (4.0%)	68 (54.0%)	53 (42.1%)	96.0%

### Mentor Feedback

Mentors who have served in different cohorts report being pleased to see how the program has grown and been refined over the years, and have provided feedback for continuous improvement as part of their surveys and through discussions with the MINDS Steering Committee. In general, mentor-reported outcomes matched the improvements perceived by the students. Although mentors tended to rate student knowledge a little lower on average than the students themselves (both pre- and post-MINDS), they did agree that their teams had improved their knowledge in all key areas, and that they had enhanced their engineering and soft skills. Like students, mentors had different experiences in different teams. Mentors reported enjoying the brainstorming process with the students and the opportunity to guide them through the different ideas and options for their design, as well as connecting them with experts in the field. Mentors reported that one of the main challenges was pushing students past the idea generation stage into making a definite choice, as well as keeping the team motivated for several months in the face of academic and personal competing interests. Overall, the mentors agreed that the MINDS program was a stimulating platform in which new ideas could be born and nurtured to maturation.

Table 6 maps the relationships between skills/learning outcomes shown in Table 5, the learning objectives in Table 1, and the associated survey questions for students and mentors.

**Table 6:** Mapping of acquired skills/learning outcomes.

Skills/learning outcomes (Table 5)	Associated learning objectives (Table 1)	Associated survey questions^†^ (5-point Likert scale)
1. Apply the three key design considerations	2. Formulate a list of key design requirements to address a healthcare problem and design a conceptual medical device solution to meet those requirements 3. Identify the market and evaluate the potential for commercialization of a medical device solution concept 4. Evaluate existing intellectual property to determine patentability of a specific design idea 5. Develop and defend a regulatory strategy and pathway to market for a novel medical device concept	(S) After the MINDS program, I have a better grasp of the 3 key design considerations (M) Do you feel that the students have a better understanding of the 3 key design considerations after completing the MINDS program?
2. Articulate key features of product	2. Formulate a list of key design requirements to address a healthcare problem and design a conceptual medical device solution to meet those requirements 6. Communicate technical information effectively in oral and written formats	(B) The MINDS program helped me (the team) learn to better articulate the key features of my (their) product
3. Articulate benefits and impact of product	2. Formulate a list of key design requirements to address a healthcare problem and design a conceptual medical device solution to meet those requirements 6. Communicate technical information effectively in oral and written formats	(B) The MINDS program helped me (the team) learn to better articulate the benefits and impact of my (their) product
4. Assess key product requirements	2. Formulate a list of key design requirements to address a healthcare problem and design a conceptual medical device solution to meet those requirements	(S) The MINDS program prepared me to be able to assess key product requirements (M) During the MINDS program, your team learned to better assess key product requirements
5. Collaborate effectively	1. Manage a collaborative long-term project with teammates and mentors from different institutions	(S) Participation in MINDS has helped me learn how to be part of collaborative projects between institutions and given me tools to collaborate more successfully (M) During the MINDS program, your team learned about distance collaboration and how to successfully coordinate people working on a project
6. Improve teamwork skills	1. Manage a collaborative long-term project with teammates and mentors from different institutions	(S) Participation in MINDS has improved my teamwork skills This question focuses on student lived experience, so there is no question on the mentor survey
7. Resolve conflicts effectively	1. Manage a collaborative long-term project with teammates and mentors from different institutions	(B) Participation in MINDS has improved my (your team’s) ability to prevent or resolve conflicts that may arise in a product development team, or to learn how to work with others who have different working styles
8. Communicate effectively	1. Manage a collaborative long-term project with teammates and mentors from different institutions 6. Communicate technical information effectively in oral and written formats	(B) Participation in MINDS has helped to increase my (your team’s) communication skills (both communicating with team members and mentors; as well as creating a report that communicates your ideas clearly)
9. Explain technical subject matters to others	6. Communicate technical information effectively in oral and written formats	(S) Participation in MINDS has helped to increase my confidence to explain technical subject matters to others, either in writing or orally This question focuses on student self-confidence, so there is no question on the mentor survey
10. Understand role of key personnel in product development	3. Identify the market and evaluate the potential for commercialization of a medical device solution concept 4. Evaluate existing intellectual property to determine patentability of a specific design idea 5. Develop and defend a regulatory strategy and pathway to market for a novel medical device concept	(B) The MINDS program helped me (your team) to identify and understand the roles of key personnel (patent attorney, technology transfer office personnel, innovation officer, etc.) who can support product design and development
11. Understand intellectual property / patent process	4. Evaluate existing intellectual property to determine patentability of a specific design idea	(S) The MINDS program helped me to understand the concept of intellectual property and the patent process (M) During the MINDS program, your team expanded their knowledge of intellectual property and the patent process
12. Understand development process from idea to virtual prototype	2. Formulate a list of key design requirements to address a healthcare problem and design a conceptual medical device solution to meet those requirements 3. Identify the market and evaluate the potential for commercialization of a medical device solution concept 4. Evaluate existing intellectual property to determine patentability of a specific design idea 5. Develop and defend a regulatory strategy and pathway to market for a novel medical device concept	(B) The MINDS program increased my (the team’s) knowledge of how a product is developed from idea to virtual prototype
13. Identify limitations of a product	2. Formulate a list of key design requirements to address a healthcare problem and design a conceptual medical device solution to meet those requirements 3. Identify the market and evaluate the potential for commercialization of a medical device solution concept 4. Evaluate existing intellectual property to determine patentability of a specific design idea 5. Develop and defend a regulatory strategy and pathway to market for a novel medical device concept	(S) The MINDS program helped me to identify potential limitations of a product being designed (M) During the MINDS program, your team improved their skills at identifying potential limitations of a product being designed

^†^Survey questions that had almost identical wording are indicated by (B), meaning “both students and mentors”, with any minor difference in wording shown within the text in parentheses. Questions that were either worded differently or were unique to one of the two groups are indicated by either (S) for students or (M) for mentors

### Themes from Student Open-Ended Comments

Students also provided many open-ended comments in their exit surveys. The question prompts were: “What aspect of your MINDS Scholar experience did you enjoy the most?”, “What aspect of your MINDS Scholar experience was the most challenging?”, “Do you have any comments on how the MINDS workshop has impacted your engineering skills?”, “Do you have any comments on how the MINDS workshop has impacted your career goals?”, and “Please provide any additional comments”. Yearly, the survey comments from both students and mentors were reviewed and utilized for continuous improvement. For instance, the inclusion of periodic mini-deliverables, the progression to a two-mentor model, as well as the introduction of a closing event where the different projects were showcased so all students could learn more about each other’s projects were all results of this continuous feedback process. Students were generally very grateful to have participated in the program and very vocal about how it is a unique opportunity, with comments such as “This program is fabulous!! It needs more recognition and promotion around the BME community. I think it would be a fun idea to have these presentations/reports be accessible to more BME students.” Comments from 2015 to 2022 were analyzed for overall themes. There were 7 main themes: learning by doing (where students described the active, hands-on approach of the program), learning outside the classroom (where students commented on how the experience was different from their regular curricular activities), learning from challenges (where students spoke about how they encountered barriers and how they overcame them), teamwork and networking (where students discussed team dynamics and the connections they established within and beyond teams), communication (where students expressed how the program had impacted their ability to disseminate information or to communicate with others), value of mentorship (where students emphasized how mentorship impacted their experience), and impact on career/carryover to professional skills (where students commented on how the experience had changed their career plans, or how it had helped them grow as professionals). Supporting illustrative quotes are provided in Table 7.

**Table 7:** Themes from student open-ended comments.

Themes	Sample quotes
*Learning by Doing* This theme is connected to objectives 1, 2, 3, 4, 5 and 6; and to skills 1, 4, 10, 11, 12, 13	• I most enjoyed conducting research on the design considerations. For my team, I was focused on the regulatory strategy and while researching a strategy to bring our product to market I went through countless pages of the FDA website to see where our product would fit. I enjoyed this process because I felt that I was learning vital information about the product development process • I enjoyed the design of the product the most. I liked the creative freedom we generally had and I enjoyed coming up with an idea and seeing it modeled and brought into a visual prototype beyond a simple drawing. Seeing the idea blossom from a crude sketch into an entire 3D model was my favorite part of this experience • I enjoyed exploring the prior art for my device and delving into the background information of our health care need. It was all new information to me, and I am glad that I learned about it • The iterative design process was emphasized, and the constant review of work was invaluable • I enjoyed the whole process of creation of the novel medical device. For the first time in a long time, researching was fun. Getting to develop a product was something I will be doing more of • The MINDS program allowed me to become a part of all the aspects of the design process, all the way to steps for regulatory and intellectual property. This helped me realize that as an engineer I need to not only make my product work effectively, but also make it so that it complies with the proper standards and regulations • I enjoyed gaining more experience with conducting patent searches and learning a bit more about how the 3 key design considerations impacted the product development process • I enjoyed enhancing my knowledge of the key design considerations by performing research into how these can be applied to my design
*Learning Outside the Classroom* This theme is connected to objectives 1, 2, 3, 4, 5 and 6; and to skills 1, 4, 5, 10, 11, 12, 13	• The MINDS workshop has taught me that one must take the three design considerations into account when designing a product. A majority of my engineering classes have focused more on the technical aspects of designing a product and have not taken these considerations into account. However, I now realize that these aspects are incredibly important when attempting to develop a new product • I enjoyed having a project that is not class related, there is no grade that comes with this, allowing some liberty and time to work on the project • While most of my academic classes have been focused on scientific content, this MINDS opportunity exposed me to the important considerations that go into engineering projects beyond the technical work. Design is so much more than simply creating a device-- design is composed of a complex web of market, regulatory, and intellectual property factors that come into play • I liked the foundational knowledge that came with it. Our BME classes this year were not well suited to engaging in the design process itself, so it was good to have that before we do so in the coming semester
*Learning from Challenges* This theme is connected to objectives 1 and 6; and to skills 5, 7, 8	• The most challenging part of the program was learning about the regulatory process as we had little knowledge about this process from our engineering classwork • My group had some issues getting all of our group members to contribute to the work that was distributed evenly but we were able to help other members out who were struggling • The most challenging part of the experience for me was the presentation portion. Once we had the project done, it was time to show our mentors what we'd come up with […] I remember going into a lot of detail rather than keeping things straight to the point, something the mentors noted in their feedback. Through that trial, I was able to learn how to present the material in a way that wasn't full of info but rather in a way that would show the practicality or feasibility instead. This was a shift from my usual way of presenting and it was difficult to adjust, but in the end, I was able to learn and grow from it • There really was not much that I did not enjoy. Of course, I occasionally did not want to join meetings or do research out of my own laziness or need to focus on other things. The most challenging part for me, though, was working on a project that I probably never would have been interested in or done research on without this team. Coming into this, I had no idea what biomarkers and paper-based assays were, and have little interest in that field, so getting myself to like this project was challenging at first. However, I am now doing my technical writing paper on paper-based microfluidics, thanks to our project • The most challenging aspect of the MINDS Scholar experience was staying on top of the work when my schoolwork got heavy. However, while this was challenging, I believe it helped me to better my time management skills • It was very difficult in the beginning to find a time when my entire team was available to meet. Once we determined our meeting time, though, we were able to stick to this throughout the program. It was also challenging to balance the competing events in each team members' life; each of us had various courses, projects, and commitments that had to be considered in planning how we would complete the project. At some time throughout the program, we all had to pick up some slack to help another team member get through a particularly busy time. Thankfully, each team member took the project seriously and did their best to contribute their share of the work
*Teamwork and Networking* This theme is connected to objectives 1 and 6; and to skills 5, 6, 7, 8	• I really enjoyed working with the mentors and students from other colleges in the same program as me. It was interesting to see the different ideas and perspectives we all had and to help others along the process for the people who had more experience • MINDS did well in pushing students to network with others outside of their comfort zone, building connections between universities that may be beneficial for one's future career • I really enjoyed engaging and getting to know my team. We came from such broad backgrounds and team meetings were always productive and enjoyable • I enjoyed getting connected with students and mentors across the nation. It definitely allowed for a unique networking opportunity • I enjoyed the team meetings and being able to communicate and connect with people I otherwise would never even have met. Some of my teammates were so hardworking and motivated, that it inspired me to be a harder worker and excited me to contribute to the project. So many new ideas circulated our meetings and it was just so great hearing fresh new perspectives • I really loved the final zoom gathering we participated in. It was interesting to see the other projects that were completed and the varying approaches everyone took. One of the groups even did their project on my original idea! The overall collaboration and consideration of different ideas for medical devices was extremely interesting and fun to experience
*Communication* This theme is connected to objective 6; and to skills 8 and 9	• Working on a project with people from different universities has helped me develop my communication skills to allow me to explain ideas more clearly and concisely • I really learned a lot and I am really thankful for this program. It has taught me considerations of design that are not stressed greatly in my coursework, and it helped me polish intangible communication skills within a design team over long distances • Learning how to communicate with a design team over long distances was very beneficial and revealed to me the challenges of organizing meetings over time zones and careers • The most challenging part of the experience was scheduling and coordinating with team members. At times, due to various events happening in our lives, we would fall slightly out of pace with each other. Whilst this wasn't desirable it encouraged us to focus on developing our communication and time management skills throughout the program, improving them with each meeting and deliverable • MINDS exposed us to the entire design process and helped us to negotiate with the other team members when the proposed design specifications conflicted
*Value of Mentorship* This theme is connected to objective 1, and to skill 5	• The mentors provided great insight into the feasibility of designs and worked to guide ideas in a manner that allowed us as students to solve problems but gave a little "nudge" in a path that would ultimately lead us to what I would call success • I loved the collaborative environment and the resources the mentors provided us with. I learned the most when our mentors brought in experts in certain fields related to designing a product • We personally interviewed several anesthesiologists/surgeons from across the globe, including, Peru, Brazil Dubai, India, US, Venezuela, and Spain to comprehend the prevalence of the problem worldwide and understand the market need. With the assistance of our mentors, we learned more about the intellectual property, regulatory pathway, reviewed relevant patents and are currently in the process of patenting the idea of our device. [...] All of this helped me to enhance my skills in designing and developing a medical device [...] • It has connected me with great mentors that I could use in future endeavors. It has also motivated me to seek out design competitions, where I can apply the skills that I learned over the course of the MINDS program • It was an honor to work under my mentors - they deserve heaven and more!! I can proudly mention that this program greatly helped me with my personal and academic goals, and now I'll be able to show that at MIT over the summer! Thank you for all you do for others!! • I really enjoyed having two mentors to answer any questions we have. They are extremely helpful and are great resources for various questions, even outside the MINDS program • I enjoyed the feedback from my mentors because they brought up points we did not consider • Our mentors pushed us to meet people and ask them questions. Those interviews with CEOs and other people in the industry were the most insightful • I greatly appreciated the mentorship we received. It was really interesting to hear from people who were already in industry or academia because they were able to provide different viewpoints on certain ideas based on their experience • I most enjoyed meeting with faculty and field professionals to learn about the different design considerations, as well as receiving feedback and advice from our mentors. Receiving firsthand information from these professionals, and receiving direct feedback and assistance on our design project, was very helpful
*Impact on Career / Carryover to Professional Skills* This theme is connected to objectives 1, 2, 3, 4, 5 and 6; and to skills 1 through 13; as well as to the quantitative survey question on career goals.	• The MINDS program helped me gain valuable professional skills to help me in my future engineering career. […] I now have direct experience in seeing a design project through from the initial idea stages to a detailed, realistic plan of execution. These skills that I have learned are very valuable for me as I graduate and look to join the engineering community • MINDS has encouraged me to consider future options in product design and development, as well as inspired me to consider starting small companies surrounding my project ideas following graduation. I am definitely more prepared to work in industry after I graduate • Prior to MINDS, I was on the pre-med track for sure, but now after having enjoyed coming up with a design for a new product and overall genuinely being curious as to where we can take our design further, I am considering whether industry or grad school may also suit my interests • Prior to MINDS, I was strongly set on getting a Master's Degree because I was very intimidated about entering the workforce. However, having completed this project I feel that I have a stronger foundation for the design and work process so that I would be comfortable entering industry immediately • This allowed me to dive deeper into regulatory strategy and FDA regulations which I see myself pursuing more in the future. I plan to graduate with my six-sigma black belt which will allow me to pursue a career in quality engineering. In researching the FDA more in depth, I see a true career path for myself involving this type of research and application • We learned a great deal regarding the process of designing and developing a medical device and I'm sure many of the practices we used for our project will translate well towards the rest of our undergraduate and graduate careers • It has helped my technical writing skills and critical thinking for finding unmet needs in the market • MINDS helped me improve my problem-solving and design skills

## Discussion

The major contribution of this paper is the introduction of the MINDS program as an extra-curricular design program that connects students and faculty across biomedical engineering departments. The interuniversity experience uniquely exposes students to different learning environments (i.e., universities, areas of the country) and serves to build confidence by expanding advanced engineering skill sets. The diversity of the learning environment is supported by the number of student host universities. Student participants create a novel product idea, describe how they used the key translational design considerations in the product design process, and will be able to state the features, benefits, and impacts of their products through production of a promotional presentation for their product. In addition, MINDS introduces students to project management and engineering soft skills through a unique real-life experience. The MINDS program expands the breadth of extracurricular EL models with an innovative approach highlighted by the inter-university student teams, interaction of students across freshman, sophomore, junior, and senior levels, and the involvement of academic and industry mentors. The peer-to-peer active learning model promotes the sharing of environments and may in turn help build confidence. This speculation is supported by the observed learning impacts on the students regardless of their level and motivates exploration of similar types of peer-to-peer interactions. Additionally, mentors identify the opportunity to interact with students and with other mentors from different universities as a valuable aspect of MINDS. A clear student benefit of the program outside of experiencing the design process is forming relationships with the mentors that they would not otherwise have had, being at different universities. These relationships often result in requests for recommendation letters, additional mentoring, and direct impacts on student careers.

The MINDS program was a first team-based design experience for many of the students and further supports the value of EL which was recognized by student appreciation of learning by doing, learning from challenges, teamwork and networking, communication, value of mentorship, and the program’s impact on their career. Although our thematic analysis is limited by its purely qualitative approach, there are clear connections between identified themes from student comments and acquired skills, as demonstrated by the quantitative portion of the analysis. Comparisons of pre- and post-experience surveys support student learning in knowledge of key areas: product development, regulatory strategy, intellectual property protection, the patent process, and marketing considerations. There were significant increases (p < 0.001) in perceived knowledge in all key areas targeted by the program. In addition, our results support an impact of experiential learning on self-reported skills including a variety of product design-related requirements and the ability to collaborate and communicate effectively. Only two out of thirteen skills fell below 90% for responses of agreed or highly agreed. These were related to resolution of conflicts (67.5%) and the roles of personnel in product development (69.1%). Regarding the roles of personnel in product development, this was not generally emphasized in the program, and exposure to this aspect depended highly on the outreach activities that were freely initiated by students and their mentors, thus varying from team to team. Regarding conflict, some students pointed out that they did not feel conflict resolution was a focus of their experience, because they did not have conflicts in their team, so they did not perceive that their skills in this area had necessarily changed. During the initial workshop, mentors emphasize the importance of communication and identify that conflicts are to be expected. The requirement of deliverables serves to hold students accountable and a focus on making design decisions based on data-supported rationale encourage discussion of known information rather than student opinions. This approach helps to minimize conflicts, yet conflicts of course do occur. The meeting formats complemented by the sharing of personal experiences by students and mentors (i.e. getting to know each other) encourage teamwork and communication when conflicts do arise. Most importantly, mentors use conflicts as a teaching moment, focus on solutions identified by the entire team, and ensure students that their experiences will be OK moving forward. Another thing mentors do is encourage constructive approaches to decision-making very early by recommending systematic approaches to selecting the project idea from the list proposed by the team. This sets a tone that teams tend to carry forward in making future decisions.

The interuniversity interactions serve to build skills in long-distance collaboration. At the start of the MINDS program 7 years ago, this type of interaction was not common. In early year surveys, numerous students mentioned long-distance collaboration as an important professional development outcome from their participation in the program, but also as a skill with a steep learning curve. During and post pandemic, remote coordination has become common and a critical soft skill. Thus, the changing academic and professional environment has increased the impact of collaboration on professional skills. Recently increased exposure to online collaboration formats as part of regular curricula has improved the ability of the last two cohorts to work effectively in a remote environment, however, student challenges related to communication, time management and teamwork challenges still remain as students adapt to an environment where they must take initiative in their own learning.

MINDS being an optional program means that, compared to the general student population, participants are generally more self-motivated to learn marketable skills, satisfy expectations of teammates and mentors, expand their professional network, and complete a professional activity for their portfolio. Still, sustaining motivation for an extracurricular project over the span of 6 months, while managing time in the face of competing school and personal responsibilities and conflicting schedules, continues to challenge teams over the years. Historically, 4 out of 135 total students who were initially selected for MINDS disengaged at some point in the process and did not complete the program. While we do not always know for sure the reasons, we speculate some of these students realize the extracurricular and long-term commitment and are unable to continue. However, the reasons are not always negative or easily predicted—for example, at least one of these students could no longer participate due to the starting of a full-time job. Overall, the high rate of program completion reflects an appreciation of the experiential value by the student participants. Team dynamics and team and program support are critical external factors that can impact a student’s ability to complete the program despite other challenges.

One of the critical roles of MINDS mentors is guiding team dynamics and project management. These are especially important given the different experience levels of the students. As an example of how team meetings are guided to encourage peer to peer interactions and workload distribution, mentors emphasize the importance of deliverables. During each meeting, students distribute deliverables to be shared during the next meeting. These deliverables are typically PowerPoint slides and CAD files, and these represent information or tasks necessary for the next step in the design process. Another complementary strategy used by mentors is to review the timeline and upcoming milestones so that students recognize how their contributions build upon each other. With these strategies being noted, the challenge of workload distribution is not straightforward to address and common to most teams. However, the requirement of student assigned deliverables helps promote a feeling of accountability and peer interactions. While we do not explicitly list peer mentoring as an expectation at the start of the program, mentors look for ways to encourage peer to peer mentoring based on different backgrounds and knowledge on the team. As an example, one of the students in a recent cohort had taken a course on medical device commercialization, and she provided constructive advice and feedback to teammates who were working on regulation and marketing.

**Figure 3:**
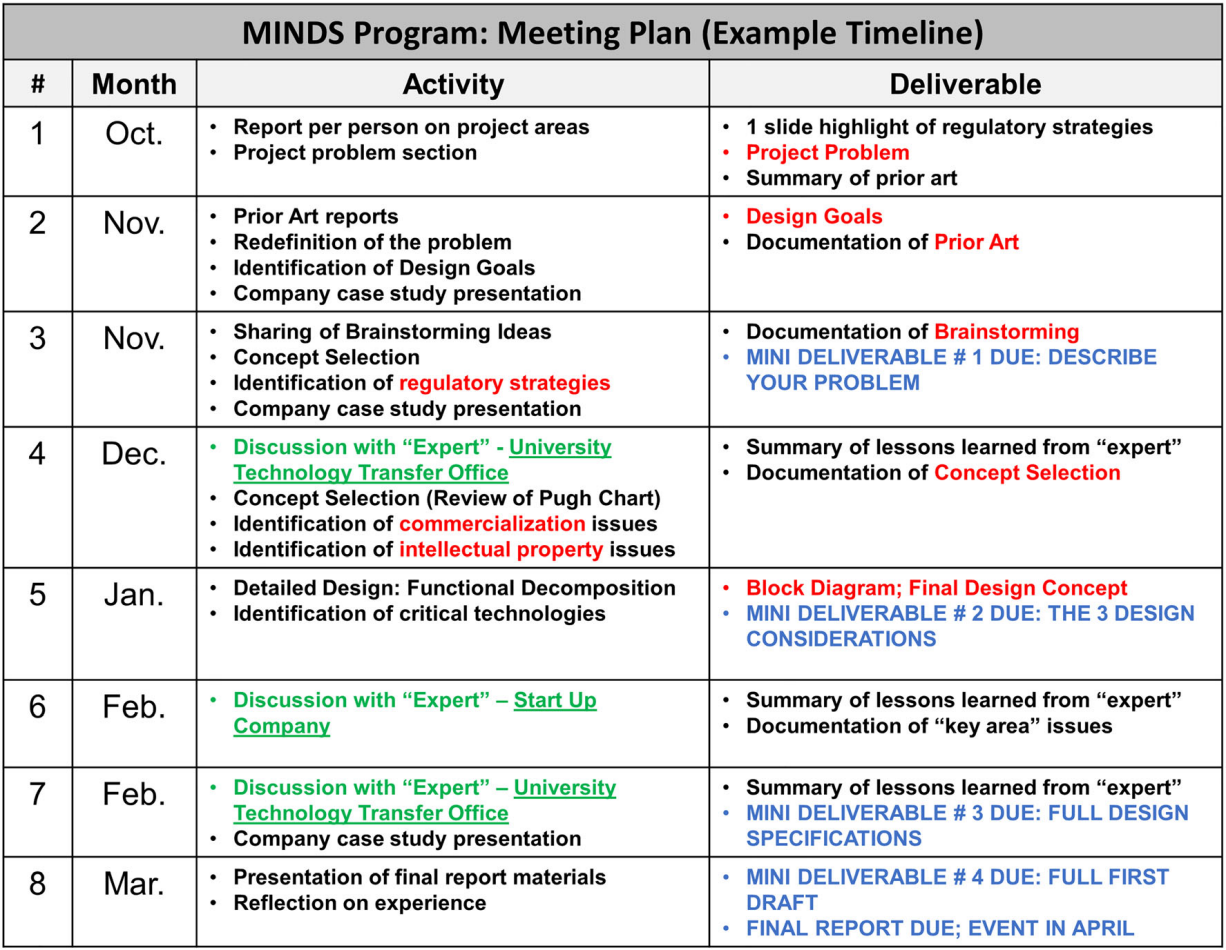
MINDS program example meeting timeline. Over the 6-month timeline, students are guided through the design process according to design steps and related mini-milestones.

**Figure 4:**
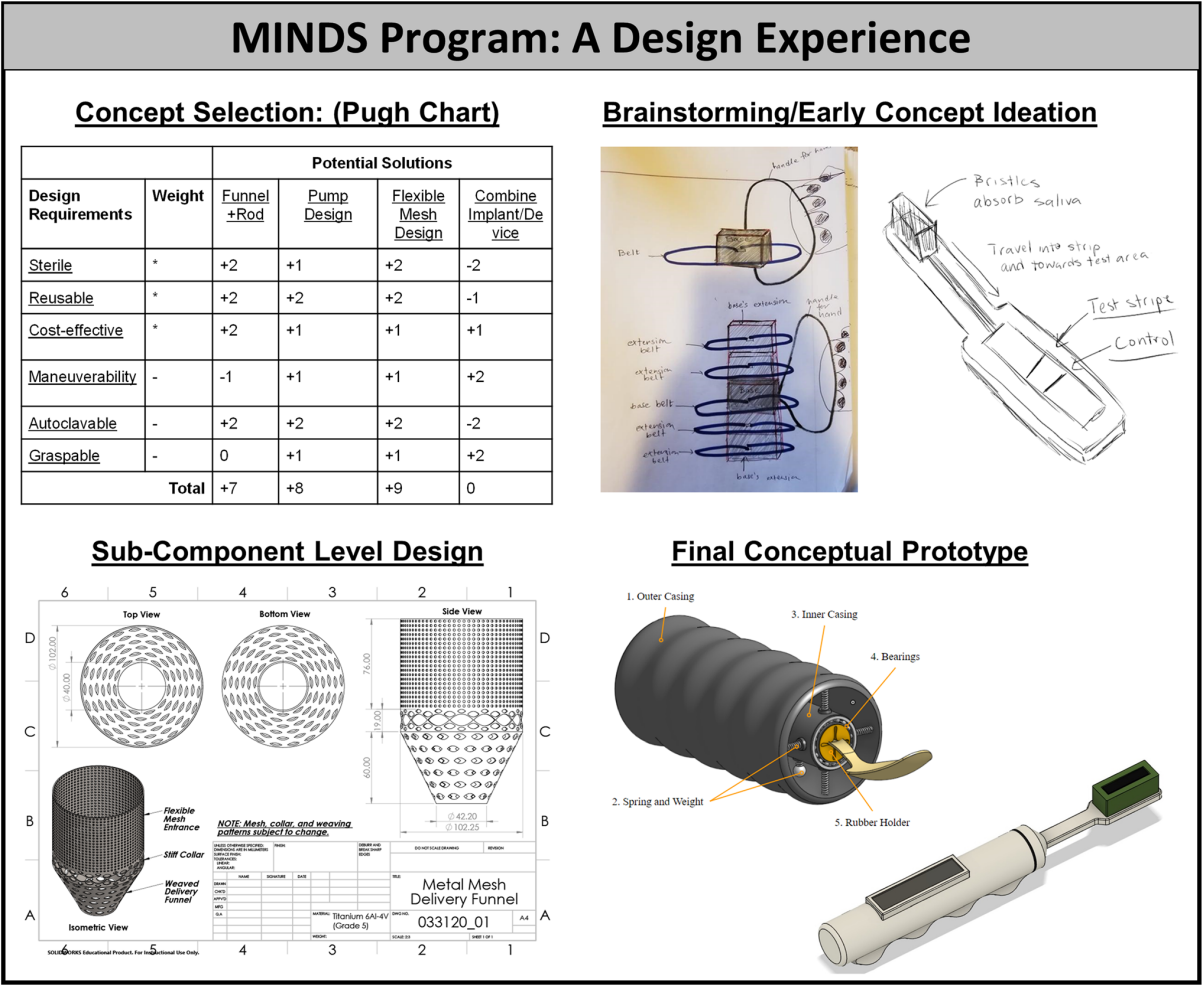
Representative MINDS program student deliverables. Students report on specific design steps including concept selection, prior art, brainstorming, component level design, and conceptual prototyping. The iterative student deliverables support student experiential learning of the design process.

As is the case for any EL program, student impact can be indeed appreciated through the challenges, and another consistent challenge for students is making decisions. In particular, teams often struggle with settling on a design topic during the first stages of the project. Mentors must guide students to make decisions through the design process by stimulating questions such as... What problem do we choose? What design concept should we select? How do we select design details? To provide adequate guidance without stifling student ownership of the learning process, a goal of the mentors is to push students along and emphasize rationale-based iterations (Fig. 3). The MINDS program’s meeting plan timeline and mini-deliverables offer a tool to enforce project management practice. Pushing along the design process in turn results in student prototype development (Fig. 4). Accomplishment of each mini-milestone encouraged student confidence in making the next decision, and most importantly in making mistakes and learning how to utilize each team member’s strengths and weaknesses. Based on student feedback, future MINDS program experiences will include increased peer feedback and sharing across teams. Students have commented that their confidence in decision-making is increased by understanding or appreciating the challenges experienced by other teams. Often the challenges are common to different teams, and recognition of this also serves to increase student confidence. Finally, an important aspect of the program is the preparation of the final report which includes a reflection-on-action process. Students are cued to reflect on accomplishments and challenges during the 6 months of the program, how they worked as a team, how they utilized internal and external resources, and how they can extrapolate what they have learned to other experiences. Students often do not fully realize how much they have accomplished until it is all put on paper and later shared with others. The reflection portion of the report is a crucial part of the learning process that cements student internalization of soft skills as well as realization of their collaborative growth.

An interesting aspect to contemplate as the program continues to grow is that while students are exposed to patents and the role of intellectual property in commercialization of a product or starting a company, the current focus of the program does not formally address the students’ roles as inventors. Team mentors are coached to guide progression of the student teams through the design process rather than influence the idea generation. With this being noted, an opportunity moving forward is to leverage the experience to make students aware of their roles as inventors. Based on experiences at our universities, students own their ideas. So, we expect that the students in the program own their ideas and the universities do not have ownership. Going forward, a formal definition of student intellectual property needs to be developed in consultation with university technology transfer offices to explore how this design experience should be best handled.

In summary, through participation in MINDS, students learn how to incorporate assessments of marketability, intellectual property protection, regulatory strategy, and end-user considerations into their design process since these are crucial to successful translation. As these topics are often not covered in rigorous detail in the standard engineering curriculum or not covered multiple times in a BME curriculum, the program supplements or reinforces classroom learning. The MINDS program also provides an opportunity for students to develop soft skills, such as conflict resolution, time management, and effective communication through the challenges of inter-institutional collaboration. After 7 years of offering the MINDS program, student feedback supports that the extra-curricular EL opportunity additionally has resulted in the discovery of new career options and improved communication skills. An additional positive aspect of the MINDS program that we look forward to leveraging more in future years is the ability to connect students from smaller or less-research active universities with students in larger departments, and to provide them with further exposure to people in the BME field and the many types of career tracks that are available. Our modular foundation allows for future program expansions to increase broader impact, and as the program continues to grow, there are opportunities for further exploring program outcomes across institution types (size, public/private, etc.) and/or specific participant demographics. Another aspect we would like to explore is longer-term curricular impact, in particular surveying graduates of the MINDS program 1 year after their experience in order to assess the effect of MINDS on curricular components such as the senior design course or other design-related courses. The success of the MINDS program demonstrates the value of innovative EL approaches and a role for national societies in enhancing student teaching. We hope that the MINDS program can inspire other curricular and extracurricular activities that maximize student involvement in experiential learning, collaboration, and discovery.
